# Genome-wide association study of frontotemporal dementia identifies a *C9ORF72* haplotype with a median of 12-G4C2 repeats that predisposes to pathological repeat expansions

**DOI:** 10.1038/s41398-021-01577-3

**Published:** 2021-09-02

**Authors:** Lianne M. Reus, Iris E. Jansen, Merel O. Mol, Fred van Ruissen, Jeroen van Rooij, Natasja M. van Schoor, Niccolò Tesi, Marcel J. T. Reinders, Martijn A. Huisman, Henne Holstege, Pieter Jelle Visser, Sterre C. M. de Boer, Marc Hulsman, Shahzad Ahmad, Najaf Amin, Andre G. Uitterlinden, Arfan Ikram, Cornelia M. van Duijn, Harro Seelaar, Inez H. G. B. Ramakers, Frans R. J. Verhey, Aad van der Lugt, Jurgen A. H. R. Claassen, Geert Jan Biessels, Peter Paul De Deyn, Philip Scheltens, Wiesje M. van der Flier, John C. van Swieten, Yolande A. L. Pijnenburg, Sven J. van der Lee

**Affiliations:** 1 Alzheimer Center Amsterdam, Department of Neurology, Amsterdam Neuroscience, Vrije Universiteit Amsterdam, Amsterdam UMC, Amsterdam, the Netherlands; 2 Department of Complex Trait Genetics, Center for Neurogenomics and Cognitive Research, Amsterdam Neuroscience, Vrije University, Amsterdam, the Netherlands; 3 Department of Neurology and Alzheimer Center Erasmus MC, Erasmus University Medical Center, Rotterdam, the Netherlands; 4 Department of Clinical Genetics, Amsterdam UMC, Amsterdam, the Netherlands; 5 Department of Epidemiology and Data Science, Amsterdam UMC, Vrije Universiteit Amsterdam, Amsterdam Public Health research institute, Amsterdam, the Netherlands; 6 Section Genomics of Neurdegenerative Diseases and Aging, Department of Clinical Genetics, Vrije Universiteit Amsterdam, Amsterdam UMC, Amsterdam, the Netherlands; 7 Delft Bioinformatics Lab, Intelligent Systems Department, Delft University of Technology, Delft, the Netherlands; 8 Department of Sociology, VU University, Amsterdam, the Netherlands; 9 Department of Psychiatry & Neuropsychology, Alzheimer Center Limburg, School for Mental Health and Neuroscience, Maastricht University, Maastricht, the Netherlands; 10 Department of Neurobiology, Care Sciences and Society, Division of Neurogeriatrics, Karolinska Institutet, Stockholm, Sweden; 11 Department of Epidemiology, Erasmus Medical Centre, Rotterdam, the Netherlands; 12 Department of Internal Medicine, Erasmus University Medical Center, Rotterdam, the Netherlands; 13 Department of Radiology & Nuclear Medicine, Erasmus University Medical Center, Rotterdam, the Netherlands; 14 Radboudumc Alzheimer Center, Department of Geriatrics, Radboud University Medical Center, Nijmegen, the Netherlands; 15 Department of Neurology, UMC Utrecht Brain Center, Utrecht, the Netherlands; 16 Department of Neurology and Alzheimer Center Groningen, University Medical Center Groningen, Groningen, the Netherlands

**Keywords:** Genetics, Diagnostic markers, Psychiatric disorders

## Abstract

Genetic factors play a major role in frontotemporal dementia (FTD). The majority of FTD cannot be genetically explained yet and it is likely that there are still FTD risk loci to be discovered. Common variants have been identified with genome-wide association studies (GWAS), but these studies have not systematically searched for rare variants. To identify rare and new common variant FTD risk loci and provide more insight into the heritability of *C9ORF72-*related FTD, we performed a GWAS consisting of 354 FTD patients (including and excluding *N* = 28 pathological repeat carriers) and 4209 control subjects. The Haplotype Reference Consortium was used as reference panel, allowing for the imputation of rare genetic variants. Two rare genetic variants nearby *C9ORF72* were strongly associated with FTD in the discovery (rs147211831: OR = 4.8, *P* = 9.2 × 10^−9^, rs117204439: OR = 4.9, *P* = 6.0 × 10^−9^) and replication analysis (*P* < 1.1 × 10^−3^). These variants also significantly associated with amyotrophic lateral sclerosis in a publicly available dataset. Using haplotype analyses in 1200 individuals, we showed that these variants tag a sub-haplotype of the founder haplotype of the repeat expansion that was previously found to be present in virtually all pathological *C9ORF72* G_4_C_2_ repeat lengths. This new risk haplotype was 10 times more likely to contain a *C9ORF72* pathological repeat length compared to founder haplotypes without one of the two risk variants (~22% versus ~2%; *P* = 7.70 × 10^−58^). In haplotypes without a pathologic expansion, the founder risk haplotype had a higher number of repeats (median = 12 repeats) compared to the founder haplotype without the risk variants (median = 8 repeats) (*P* = 2.05 × 10^−260^). In conclusion, the identified risk haplotype, which is carried by ~4% of all individuals, is a major risk factor for pathological repeat lengths of *C9ORF72* G_4_C_2_. These findings strongly indicate that longer *C9ORF72* repeats are unstable and more likely to convert to germline pathological *C9ORF72* repeat expansions.

## Introduction

Frontotemporal dementia (FTD) is the second-most common cause of early onset dementia (3.5–15 per 100,000 in <65 years), leading to a spectrum of clinical syndromes associated with frontal and/or temporal neuronal loss [1, 2]. Clinically, FTD can be classified into the behavioral variant (bvFTD) and the language variants semantic dementia (SD) and progressive non-fluent aphasia (PNFA) [3]. FTD is associated with motor neuron disease (FTD-MND) in 10% of all cases [4]. Currently, no treatment options are available for FTD. To identify potential treatment targets, an understanding of the underlying genetic etiology of FTD is highly needed.

Genetic factors play a major role in FTD; up to 40–50% of all FTD patients have a positive family history for dementia [5, 6]. Mutations that cause autosomal dominant FTD have been identified in microtubule associated protein tau (*MAPT*) [7], progranulin (*GRN*) [8], and the chromosome 9 open reading frame 72 (*C9ORF72*) G_4_C_2_ hexanucleotide repeat expansion [9, 10]. While familial mutations account for ~30% of FTD cases, the majority of FTD is multifactorial and polygenic in nature [11]. Previous genome-wide association studies (GWAS) on FTD have identified only a handful of common genetic risk variants for FTD with small effects on developing disease [12–15]. As the majority of sporadic FTD cannot be genetically explained yet, it is likely that there are still FTD risk loci to be discovered.

Rare genetic variants (minor allele frequency (MAF) ≤ 5%) often have stronger associations with disease than common genetic variants, but reliable imputation of rare genetic variants with widely used reference panels is challenging [16]. The Haplotype Reference Consortium (HRC) allows imputation of genetic variants with a MAF up to 0.001 [17, 18]. Performing a GWAS on FTD using the HRC panel as reference panel may aid in identifying rare risk variants for FTD, thereby improving insights into the genetic etiology of FTD.

To identify rare and common variant FTD risk loci and provide more insight into the pathogenesis and heritability of *C9ORF72-*related FTD, we performed a GWAS study in a cohort of Dutch FTD patients and control subjects, using the HRC panel as reference panel.

## Methods

### Study sample genome-wide association study

We performed a genome-wide association analysis in which we compared genotype data of 354 FTD patients (8%, *N* = 28 with pathological *C9ORF72* repeat length) from three cohorts with 4209 control subjects from seven cohorts. Table S1 presents a brief description of the contributing cohorts. FTD was diagnosed according to diagnostic guidelines for FTD [3, 19]. Clinical subtypes of FTD (i.e., bvFTD, SD, PNFA, and FTD-MND) were available for 311 patients from the Amsterdam Dementia Cohort (ADC). Replication analyses were performed using data from the Erasmus Medical Center and an independent sample of the LASA study, including 281 FTD patients and 618 control subjects [20, 21].

All participating studies were approved by their respective Medical Ethics Committee (Table S1). Informed consent, either from the patient or from the legal representative, was obtained from all participants.

### Genotyping and imputation

The discovery cohorts were genotyped on the Illumina Genome Screening Array (GSA, GSAsharedCUSTOM_20018389_A2) v1, human genome build 37. Quality control prior to imputation has been described in depth elsewhere [22]. Briefly, genetic variants were excluded from analyses when they deviated significantly from Hardy–Weinberg equilibrium (*P* < 1 × 10^−6^) in the total sample of founder individuals, or had a variant call rate of <98%. Individuals with sex mismatches or an individual call rate <98% were excluded from analyses. In total, 529,668 SNPs passed QC and were submitted to the Sanger imputation server for imputation to the Haplotype Reference Consortium (HRC) reference panel (https://imputationserver.sph.umich.edu). We pre-phased with SHAPE-IT2 [23]. This resulted in the imputation of 39,131,578 variants [18, 24]. To identify ethnic outliers, a principal component analysis of ancestry (PCA) was performed (based on 1000Genomes clustering), using EIGENSOFT [25]. Individuals of non-European ancestry were excluded from analysis to account for population structure. Relatedness was assessed through identity by descent (IBS), and family relations up to second degree (IBS ≥ 0.3) were excluded. To account for population structure, PCs were calculated on genetic data prior to imputation. In the replication cohort, cases were genotyped on the GSA array and the controls on the Axiom-NL array from Affymetrix (Avera Institute for Human Genetics, Sioux Falls, SD) [26, 27]. Quality control was performed in the same way as described for the discovery dataset for cases and controls independently and frequencies of the variants were compared after imputation.

### Genotyping across the GGGGCC *C9ORF72* repeat

Allele-specific polymerase chain reaction (PCR) was performed using 0.2 mM dNTPs (Solis Biodyne), 0.05 Units HotFirePol DNA polymerase (5 U/μl Solis Biodyne), 1x Buffer B (Solis Biodyne), 2 mM MgCl2 (Solis Biodyne), 7% DMSO (Sigma Aldrich), 2 μM 6FAM-fluorescent labeled forward primer ([6FAM]ACTCGCTGAGGGTGAACAAG) and 2 μM reverse primer (TCGAGCTCTGAGGAGAGCC), and 100 ng of genomic DNA. A standard PCR cycling program (35 cycles) was used where the annealing temperature was set at 55 °C with a 1-min extension time for each cycle. Fragment length analysis was performed on an ABI 3730xl/3500 genetic analyzer (Applied Biosystems Inc., Foster City, CA, USA), and data was analyzed using GeneScan software (version 4/5, ABI). Chromatograms were scored for the number of alleles and the number of repeats. Samples that have large lengths and samples with two alleles of the same length show only one band in the allele-specific PCR. For these samples, repeat-primed PCR was performed (supplementary methods).

### Phenome-wide association studies

We conducted phenome-wide association studies (PheWAS) on the two replicated SNPs, rs147211831, rs117204439, using the ‘phewas’ function of the R-package ‘ieugwasr’ [28, 29]. Using this function, we searched traits that associate with the list of SNPs with *P* < 5 × 10^−8^ in all GWAS harmonized summary statistics in the MRC IEU OpenGWAS data infrastructure [29].

### Haplotyping of the identified risk variants for FTD and *C9ORF72* repeat lengths

To further study the relationship between the FTD risk alleles identified in the GWAS (rs147211831-A and rs117204439-C) and *C9ORF72* repeat lengths, we phased *C9ORF72* repeat lengths to haplotypes. We re-imputed chromosome 9 using EAGLE2 for pre-phasing [30]. This resulted in phased imputed genotypes in contrast to phasing with SHAPE-IT2 (which has slightly higher imputation accuracy) [23].

In accordance with previous studies, we found that the founder haplotype could be simplified to just one variant, rs3849942 (founder SNP = T) (Fig. S1) [31, 32]. Therefore, we were able to construct three SNP-haplotypes covering the *C9ORF72* gene. In short, we classified all haplotypes into the ancestral (non-founder) haplotype (rs3849942-C) and the founder haplotype (rs3849942-T). Subsequently, we split these haplotypes on having at least one risk allele (rs147211831-A and/or rs117204439-C) or no risk alleles. This resulted in four haplotype groups: ancestral non-risk, ancestral risk, founder non-risk, and founder risk haplotypes. We then mapped *C9ORF72* lengths to these haplotypes using a Bayesian classifier as described in Fig. S2 and the supplementary methods. The distribution of the *C9ORF72* lengths in the training dataset of these ancestral and founder haplotypes is presented in Fig. S3.

### Statistical analysis

Association analysis on FTD patients versus controls was performed using PLINK version 2.0 [33, 34]. We used the Firth fallback option to fit logistic regression models, adjusting for population stratification (PC1-5). This model automatically uses Firth regression if the model does not converge (e.g., mainly for rare variants). SNPs with a low imputation quality (*R*^2 ^< 0.3) and a MAF < 0.5% were excluded. In total 8,813,788 variants were analyzed. To examine whether genome-wide significant loci (*P* < 5 × 10^−8^) were driven by pathological *C9ORF72* repeat carriers, analyses were repeated excluding patients who carried a pathological *C9ORF72* repeat length or did not have *C9ORF72* lengths available (discovery *N* = 275 FTD patients/239 controls; replication *N* = 198 FTD patients/618 controls).Additional analyses were performed including age and sex as covariates. We performed a meta-analysis on genome-wide significant loci (*P* < 5 × 10^−8^) using fixed-effects model with the rmeta package [35]. Last, we stratified analyses by clinical subgroups of FTD.

Additional statistical analyses were performed using R studio (version 4.0.3, Bunny-Wunnies freak out, R Development Core team 2010). To examine the association between haplotype and *C9ORF72* repeat length, we compared *C9ORF72* repeat expansion carriership using the proportion test and *C9ORF72* repeat lengths (excluding *C9ORF72* repeat expansion carriers) between haplotype groups (i.e., ancestral non-risk, ancestral risk, founder non-risk, and founder risk haplotypes), using Kruskal–Wallis test.

## Results

An overview of sample characteristics is shown in Table S2. The discovery FTD sample included less females, was younger compared to the controls and included ~8% (*N* = 28/354) pathological *C9ORF72* repeat length carriers (9%; *N* = 25/281 in replication). Of the FTD patients with clinical subtyping available, *N* = 194 were classified as bvFTD, *N* = 74 as SD, *N* = 25 as PFNA, *N* = 18 as FTD-MND and *N* = 43 were unclassified.

### Association with FTD

**Fig. 1:**
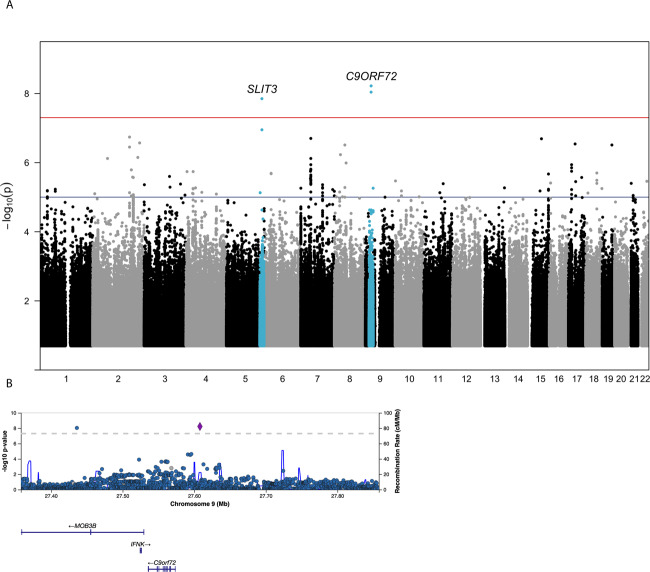
Manhattan plot and regional plot of the discovery analysis on the genome-wide association with frontotemporaldementia. **A** Manhattan plot. The discovery analysis included *N* = 354 FTD patients and *N* = 4209 controls. The genome-wide significance threshold (*p* < 5 × 10^−8^) has been highlighted in red and the suggestive significance threshold (*P* < 1 × 10^−5^) is depicted in blue. For each genome-wide significant locus, loci are named by the closest located gene. **B** Regional plot for the *C9ORF72* locus on chromosome 9. The genetic variant depicted in purple represents the strongest associated variant. Abbreviation(s) FTD: frontotemporal dementia, *C9ORF72:* chromosome 9 open reading frame 72, *SLIT3:* Slit Guidance Ligand 3.

Variants in two genomic loci were significantly associated with FTD (*p* < 5 × 10^−8^) (Fig. 1A and Table 1). No genomic loci were significant in the analysis excluding pathological *C9ORF72* repeat carriers. There was no genomic inflation in the GWAS (*λ* = 0.009) (Fig. S4).

**Table 1:** FTD summary statistics discovery and replication analysis for SNPs exceeding genome-wide significance (*P* < 5e-08) in the discovery analysis.

SNP	Chr	Bp	Closest Gene	ref/alt	% alt	Discovery				Replication				Discovery excluding				Replication excluding			
					FTD/controls									*C9ORF72* carriers^a^				*C9ORF72* carriers^a^			
						OR	Beta	SE	*P*	OR	Beta	SE	*P*	OR	Beta	SE	*P*	OR	Beta	SE	*P*
rs76679949	5	168220640	*SLIT3*	G/C	0.044/0.019	3.75	1.32	0.23	1.40 × 10^−8^	1.58	0.46	0.34	0.18	NA	NA	NA	NA	NA	NA	NA	NA
rs147211831	9	27436084	*C9ORF72*	C/A	0.032/0.009	4.96	1.60	0.28	9.18 × 10-^9^	3.95	1.37	0.42	1.05 × 10^−3^	1.87	0.62	0.44	0.15	2.33	0.85	0.51	0.09
rs117204439	9	27607973	*C9ORF72*	T/C	0.039/0.015	4.85	1.58	0.27	5.96 × 10-^9^	3.21	1.17	0.38	2.04 × 10^−3^	2.12	0.75	0.39	0.05	1.32	0.27	0.52	0.60

^a^For these analyses we excluded FTD patients with pathological *C9ORF72* repeat lengths and FTD patients without *C9ORF72* data available (*N* = 28/52 carriers/unknown in discovery, *N* = 25/58 in replication).

FTD: frontotemporal dementia, SNPs: single nucleotide polymorphisms, Chr: chromosome, Bp: base pair location, ref: reference allele, alt: alternative allele, OR: odds ratio, Se: standard error, NA: not applicable, *C9ORF72* chromosome 9 open reading frame 72*, SLIT3* Slit Guidance Ligand 3.

A single intronic variant in a locus on chromosome 5 (rs76679949), located on the *SLIT3* (Slit Guidance Ligand 3) gene, was associated with a 3.7 times increased risk of FTD (MAF-cases = 4.4%; MAF-controls=1.9%; *P* = 1.4 × 10^−8^) (Fig. S5). There were no additional variants in linkage with this variant to support the association and we were not able to replicate the association in the replication dataset (*P* = 0.18; OR = 1.58). We consider this locus a false positive finding and did not investigate it further.

The second locus on chromosome 9 contained two genetic variants that were significantly associated with FTD risk (rs117204439 and rs147211831). These two variants are located on both sides of the *C9ORF72* gene (Fig. 1B). In our data the two variants were in partial linkage (*R*^2^ = 0.041; D′ = 0.52) and 48% (43/90) of the carriers of rs117204439-C also carried the rs147211831-A allele. The most significant SNP, rs117204439, associated with a ~4.9 times increased risk of FTD (risk allele = C; MAF-cases = 3.9%; MAF-controls = 1.5%; *P* = 6.0 × 10^−9^). The second variant, rs147211831, associated with a ~4.8 times increased risk of FTD (risk allele = A; MAF-cases=3.2%; MAF-controls=0.9%; *P* = 9.2 × 10^−9^). When including the allele status of rs117204439 as covariate in the logistic regression model on FTD, the association of rs147211831 with FTD remained significant (*P* = 4.8 × 10^−3^; OR = 2.7). This was also the case for the association of rs147211831 with FTD, corrected for rs117204439 (*P* = 2.6 × 10^−3^, OR = 2.8). These observed residual associations after adjusting for the other SNP suggests that signals were driven by their shared haplotype rather than by the specific SNP. Analyses stratified by clinical subtypes of FTD showed that associations were strongest in bvFTD (OR = 5.3–5.5) and FTD-MND (OR = 13.1–16.9) (Fig. S6). Results for rs147211831 and rs117204439 were similar when repeating the analyses correcting for age and sex (Table S3).

In our independent replication datasets, both variants near *C9ORF72* significantly associated with increased risk for FTD (rs117204439 MAF-cases = 3.6%; MAF-controls = 1.5%; *P* = 2.0 × 10^−3^; OR = 3.2, rs147211831 MAF-cases = 3.1%; MAF-controls = 1.1%; OR = 3.95, *P* = 1.1 × 10^−3^) (Table 1). Associations were strongest in bvFTD (OR = 3.9-4.1) and FTD-MND (OR = 6.2–6.3) (Table S4). Meta-analysis on the discovery and replication data showed similar results (rs117204439; *P* = 5.6 × 10^−11^; OR = 4.22, rs147211831; *P* = 5.2 × 10^−11^; OR = 4.62) (Table S5). After excluding pathological *C9ORF72* repeat length carriers and FTD patients without *C9ORF72* data (*N* = 28/52 carriers/unknown in discovery, *N* = 25/58 in replication) the association was no longer significant in both the discovery (rs117204439; *P* = 0.05; OR = 2.12, rs147211831 *P* = 0.15; OR = 1.87) and the replication cohorts (rs117204439 *P* = 0.60; OR = 1.32, rs147211831 *P* = 0.09; OR = 2.33). It is unlikely that results were driven by a single Dutch family, as the haplotype is relatively common and family relations up to second degree were excluded from analyses.

### FTD risk alleles associate with amyotrophic lateral sclerosis in PheWAS

Both of the identified risk alleles for FTD showed an association with amyotrophic lateral sclerosis (ALS) (*N*_GWAS _= 12,663 ALS patients/53,439 controls) [36]. Risk allele rs147211831-A was associated with a ~1.9-fold increased risk of ALS (*P* = 2.3 × 10^−20^) and rs117204439-C with a 1.6-fold increased risk (*P* = 3.1 × 10^−14^) (Table S6). No other traits showed significant associations with the variants.

### Screening of risk SNP carriers for *C9ORF72* repeat expansions

The ADC also includes subjects diagnosed with other types of dementia and mild cognitive impairment (*N* = 2543). From these samples, we selected 58 non-related carriers of the FTD risk alleles rs117204439-C and rs14721183-A of European ancestry. We found that four of these 58 risk allele carriers had a pathological *C9ORF72* repeat expansion. The diagnoses of the patients were diverse including vascular dementia, a psychiatric diagnosis, mild cognitive impairment, and a postponed diagnosis.

### *C9ORF72* risk alleles associate with intermediate repeat length in haplotype analysis

*C9ORF72* repeat lengths were measured in a total of 1578 subjects from the ADC cohort, of whom 1327 had SNP-array data available. We excluded 104 individuals with a non-European ancestry and 23 individuals that were related (IBS > 0.2) to each other, leaving *N* = 1200 individuals for the haplotype analysis (Table S7). We attempted phasing *C9ORF72* repeat lengths to haplotypes in all *N* = 1200 participants (see Methods section and Figs. S1 and S2).

**Fig. 2:**
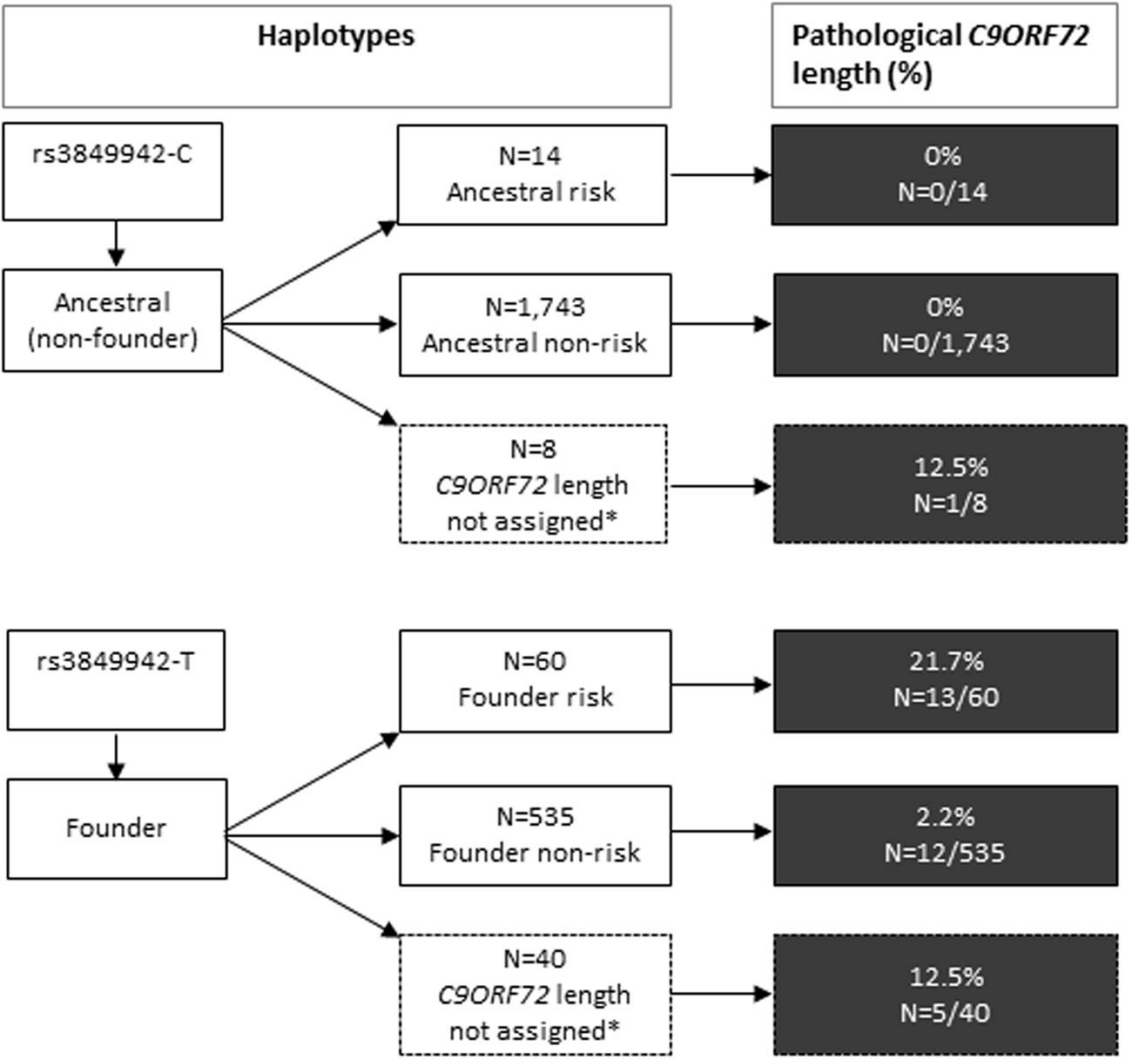
Pathological *C9ORF72* repeat lengths in *C9ORF72* haplotypes, including ancestral non-risk haplotypes, ancestral risk haplotypes, and founder non-risk haplotypes and founder risk haplotypes. Haplotypes could be mapped to *C9ORF72* repeat lengths for *N* = 1743 ancestral (non-founder) non-risk haplotypes, *N* = 14 ancestral risk haplotypes, *N* = 535 founder non-risk haplotypes, and *N* = 60 founder risk haplotypes. The founder haplotype is defined by the presence of rs3849942-C (tags ancestral allele) or rs3849942-T (tags founder allele). Risk status is defined by the presence of at least one risk allele, including rs117204439-C or rs147211831-A. *The subset off haplotypes that could not be assigned to *C9ORF7*2 lengths had a short allele with a low probability (<0.8) for the ancestral allele (rs3849942-C) and *C9ORF72* lengths differed by more than 3 repeats. Abbreviation(s) *C9ORF72*: chromosome 9 open reading frame 72.

We were able to reliably assign *C9ORF*72 lengths to the haplotype for 2352/2400 haplotypes (98%). These include 1743 (74.1%) ancestral non-risk haplotypes, 14 (0.6%) ancestral risk haplotypes, 535 (22.7%) founder non-risk haplotypes, and 60 (2.6%) founder risk haplotypes (Fig. 2).

Of all pathological *C9ORF72* repeat lengths, 96.8% (*N* = 30/31) were mapped to the founder haplotype and one was mapped to the ancestral haplotype. Of the 31 repeat expansion haplotypes, 13 (41.9%) were the founder risk haplotype, 12 (38.7%) the founder non-risk haplotype, and 6 (19.4%, 5 founder and 1 ancestral) could not be assigned to a haplotype (Fig. 2).

**Fig. 3:**
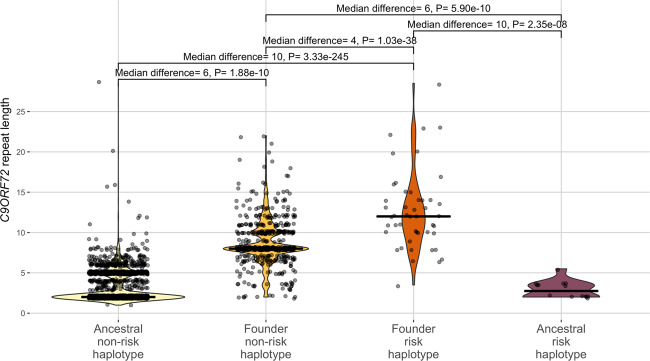
*C9ORF72* repeat length (excluding haplotypes with >30 repeats) in *C9ORF72* haplotypes, including ancestral non-risk haplotypes, ancestral risk haplotypes, founder non-risk haplotypes and founder risk haplotypes. The founder haplotype is defined by the presence of rs3849942-C (tags ancestral allele) or rs3849942-T (tags founder allele). Risk status is defined by the presence of at least one risk allele, including rs117204439-C or rs147211831-A. Abbreviation(s) *C9ORF72*: chromosome 9 open reading frame 72.

As the founder risk haplotype was much less prevalent, the *C9ORF72* repeat expansion was ~10-times more likely to be on a founder risk haplotype compared to the founder non-risk haplotype. In total, 21.7% of the founder risk haplotypes had pathological *C9ORF72* repeat lengths (*N* = 13/60) compared to the founder non-risk haplotypes (2.2%, *N* = 12/535) (*P* = 7.70 × 10^−58^) (Table S8). Next, we compared the distribution of *C9ORF72* repeats in the four haplotypes, excluding haplotypes with a pathological *C9ORF72* repeat length (>30 repeat elements). Founder risk haplotypes had a median of 12 repeat elements, which was significantly higher than the founder non-risk haplotypes (median = 8, *P* = 1.03 × 10^−38^), ancestral risk haplotypes (median = 2, *P* = 2.35 × 10^−8^) and ancestral non-risk haplotypes (median = 2, *P* = 3.33 × 10^−245^) (Table S8 and Fig. 3).

## Discussion

Our findings show that the two variants rs117204439 and rs147211831 tag a *C9ORF72* haplotype that is carried by ~4% of the population. This founder risk haplotype greatly increases the risk for a pathological *C9ORF72* repeat length, which has been associated with FTD and the related motor neuron disorder ALS. Pathological lengths were ~10-times more likely to be present on this founder risk haplotype than on the founder haplotype without the risk variants. Haplotype analyses showed that the well-known founder haplotype with at least one risk allele had a median of 12 repeats compared to a median of 8 for the founder haplotype without risk alleles. The results of this study imply that an increased number of *C9ORF72* repeat units increases the risk of conversion from a non-pathological repeat length to a pathological repeat length during parent-offspring transmissions.

Common variants at the *C9ORF72* locus at chromosome 9p21 have been identified previously as a genetic risk region for FTD and ALS [14, 15, 37–39]. Most, but not all [40], studies showed that association signals within the 9p21 region were driven by carriers of the pathological repeat length of the G_4_C_2_ repeat in the *C9ORF72* gene [9, 10, 41]. These variants tag a so-called ‘Finnish founder haplotype’ of ~200 kb [31, 42]. This haplotype has a common founder and likely originated in Northern Europe and spread from there to other regions [31, 32]. Haplotype analyses of carriers of pathological *C9ORF72* repeat lengths showed that nearly all carriers share (a part of) this haplotype. Therefore, the leading hypothesis is that pathological *C9ORF72* repeat lengths have been introduced on this haplotype into the population on multiple events due to a permissive allele [43–45]. This is a form of mutation in which repeat lengths expand within tissues [46] and during parent-offspring transmission [47], thereby predisposing to pathological repeat lengths [48, 49]. The founder haplotype had ~8 repeat units, compared to 2–4 units in the ancestral haplotype. Probably, this is the permissive allele that is associated with repeat instability. Still, it is debated whether the 8-unit repeat is more prone to repeat expansions as the inheritance of <30 repeats was found to be stable over generations [50]. On the other hand, it has been shown that pathological *C9ORF72* repeat lengths frequently vary over generations [43, 44, 51]. With our study, we add to this knowledge that a sub-haplotype of the founder haplotype with a median of 12 repeat units explains the majority of the pathological repeat lengths. This makes it plausible that the longer the G_4_C_2_ *C9ORF72* repeat is, the more likely it is that a de novo pathological expansion occurs during meiosis. Still, these expansion events must be extremely rare as the haplotype we identified is carried by only 4% of the Dutch population and by ~1–3% of all populations of European ancestry [25].

The molecular mechanisms underlying *C9ORF72* repeat instability involve DNA damage, since *C9OR72* repeats have shown to interfere with DNA replication via abnormal nuclei acid structures (e.g., the formation of G-quadruplex structures, hairpins, and R-loops) [49, 52, 53]. *C9ORF72* repeats can form abnormal nuclei acid structures with as few as four repeats and repeat instability increases with longer *C9ORF72* repeats [49]. This may explain why the founder risk haplotype (with intermediate repeats) and the founder non-risk haplotype (with a lower range of repeats) both predispose to de novo pathological repeats, but differ in the proportion of pathological *C9ORF72* repeat lengths (~21.7% and ~2.2%, respectively) [31]. Further longitudinal research in multiple generations of carriers of the identified haplotype is required to confirm the higher conversion rate to longer *C9ORF72* repeat lengths in carriers of the founder risk haplotype compared to carriers of the founder non-risk haplotype. This type of study is also required to examine whether the risk haplotype serves as a pre-mutation or as predisposing allele for further stepwise mutation. Moreover, future studies should further investigate the possibility that the *C9ORF72* region contains additional genetic and epigenetic variants conferring risk to FTD.

While the identified SNPs tagging the founder risk haplotype cannot replace the *C9ORF72* repeat length assessments itself, a potential implementation of our findings is the use of the risk SNPs as pre-screener for the presence of a pathological *C9ORF72* repeat length in large population samples with array genotype data available. We were able to identify four previously undiscovered repeat expansion carriers that had another diagnosis than FTD or ALS. This underlines the diverse clinical presentation of subjects carrying the pathological *C9ORF72* repeat expansion.

Several limitations should be taken into account. While this study provides relevant insights into the genetic architecture of FTD in populations of European ancestry, further studies are required to examine the genetic architecture of FTD in other populations – particularly because *C9ORF72* repeat lengths differ across ethnic populations [54]. Finally, we could not map all phased *C9ORF72* haplotypes to *C9ORF72* repeat lengths. Ideally, we would have used long read sequencing data to confirm the phases of these haplotypes. Nonetheless, because findings for the unmapped haplotypes are in line with the rest of our results (Table S9) we do not think that this has influenced the results.

To conclude, we identified two risk SNPs for FTD that tag a 12-repeat sub-haplotype of the 8-repeat founder haplotype, which predisposes to *C9ORF72* pathological repeat lengths. We hypothesize that the longer repeat length makes the *C9ORF72* repeat more unstable and thus more susceptible to pathological expansion. To further understand the dynamic relationship between risk founder haplotypes (with increased repeat instability) and expansions of the *C9ORF72* repeat, it is essential that our efforts will be extended using functional follow-up studies and studies over generations.

## Supplementary information

Supplemenary methods, results, figures and table captions

Supplemenary tables

## Data Availability

Codes used to generate results are available upon request.
