# Supplementary material for: Genome-wide association study of frontotemporal dementia identifies a C9ORF72 haplotype with a median of 12-G4C2 repeats that predisposes to pathological repeat expansions
Source: Transl Psychiatry. 2021 Sep 2;11:451. doi: 10.1038/s41398-021-01577-3 (PMC8413318; doi:10.1038/s41398-021-01577-3)
Supplement: Supplementary file 1 — Supplemenary methods, results, figures and table captions [file 41398_2021_1577_MOESM1_ESM.docx]

**Supplementary materials**

**Supplementary methods**

**Genotyping across the GGGGCC *C9ORF72* repeat**

For the repeat primed PCR we used Asuragen ® kit or an in-house pipeline. Conditions for Asuragen were used as outlined in the manufacturer's protocol (<https://asuragen.com/portfolio/genetics/amplidex-pcrce-c9orf72/>), and as described in depth elsewhere ^55^. Fragment length analysis was performed on an ABI 3730xl/3500 genetic analyzer (Applied Biosystems Inc., Foster City, CA, USA), and data was analyzed using GeneScan software (version 4/5, ABI).

The in-house pipeline (until 2017) used FastStart PCR Master, 7% DMSO, 1M betaine, 0.17 mM of 7-deaza-2-deoxy GTP, 0.7–1.4 μM of primer mix, 0.85mM of MgCl2 and 100ng of genomic DNA. Primers included a FAM-labelled reverse primer, one repeat-specific forward primer with an attached anchor sequence and the same anchor sequence as an independent forward primer. Cycling conditions were denaturation 95°C for 15 minutes and touchdown from 70 to 56°C with 3 min extension (70°C-2x,68°C-3x,66°C-4x,64°C-5x,62°C-6x,60°C-7x,58°C-8x,56°C-5x). Fragment length analysis was performed on an ABI 3730xl/3500 genetic analyzer (Applied Biosystems Inc., Foster City, CA, USA), and data was analyzed using GeneScan software (version 4/5, ABI). Pathological repeat lengths produce a characteristic sawtooth pattern with a 6-bp periodicity. Chromatograms were scored as mutant (sawtooth pattern) or wild type (<30 repeats). Primermix (C9 repeat_For: [6FAM]AGTCGCTAGAGGCGAAAGC, C9 repeat_Rev : TACGCATCCCAGTTTGAGACGGGGGCCGGGGCCGGGGCCGGGG, C9 repeat_Anchor: TACGCATCCCAGTTTGAGACG).

**Haplotyping of the identified risk variants for FTD and *C9ORF72* repeat lengths**

Using the rs3849942 variant as a proxy for the founder haplotype, we classified all haplotypes in ancestral (non-founder) haplotype (rs3849942-C, N=1,765, 73.5%) and founder haplotype (rs3849942-T, N=635, 26.5%). Subsequently, we split these haplotypes based on having at least one risk allele (rs147211831-A and/or rs117204439-C, N=106, 4.4%) or no risk alleles (N=2,302, 95.9%). We attempted to phasing of the *C9ORF72* repeat lengths to the SNP-haplotypes using methods described in Figure S2. For individuals in whom the difference between the *C9ORF72* repeat lengths was 3 or less, we assigned the mean length of *C9ORF72* repeats to both haplotypes, because of the error in determining the *C9ORF72* lengths. The *C9ORF72* lengths of those carrying two copies on the same haplotype, including individuals carrying two copies of the ancestral haplotype (rs3849942 C/C) or the founder haplotype (rs3849942 T/T), were used as training dataset for a Bayesian classifier. The distribution of the *C9ORF72* lengths in the training dataset of these ancestral and founder haplotypes is presented in Figure S3. For *C9ORF72* repeat length alleles of heterozygotes for the founder allele (rs3849942 C/T), we estimated the chance of an allele being on the ancestral or founder haplotype based on the Bayesian classifier. As a result, all *C9ORF72* lengths were assigned two probabilities: to be on the ancestral (rs3849942-C) and founder-haplotype (rs3849942-T). If the ancestral allele probability was over 80%, we phased the *C9ORF72* allele to the ancestral haplotype (rs3849942-C). Using this method, we fassigned 2352 of 2400 (98%) *C9ORF72* alleles to haplotypes. The subset of haplotypes that could not be assigned to *C9ORF72* lengths had a short allele with a low probability (<0.8) for the ancestral allele (rs3849942-C) and *C9ORF72* lengths that differed by more than 3 repeats. Characteristics of these unassigned haplotypes are discussed in the supplementary materials.

**Supplementary results**

Of all N=48 haplotypes for which we could not assign the three-SNP phased haplotypes to *C9ORF72* repeat lengths, N=8 were non-founder (rs3849942-C) and N=40 were founder (rs3849942-T) haplotypes. The “unsure” haplotypes had a median *C9ORF72* repeat length of 8 (IQR=8-14.75) and included six (12.5%, N=6/48) pathological repeat lengths. In the sample excluding pathological *C9ORF72* repeats, founder haplotypes (median=11) were related to an increased number of *C9ORF72* repeats compared to non-founder haplotypes (median=5) (*P*=1.03x10^-3^). The number of pathological *C9ORF72* repeat lengths did not differ between founder (12.5%, N=5/40) and non-founder haplotypes (12.5%, N=1/8) (*P*=1.00) (Table S9).

**Supplementary figures**


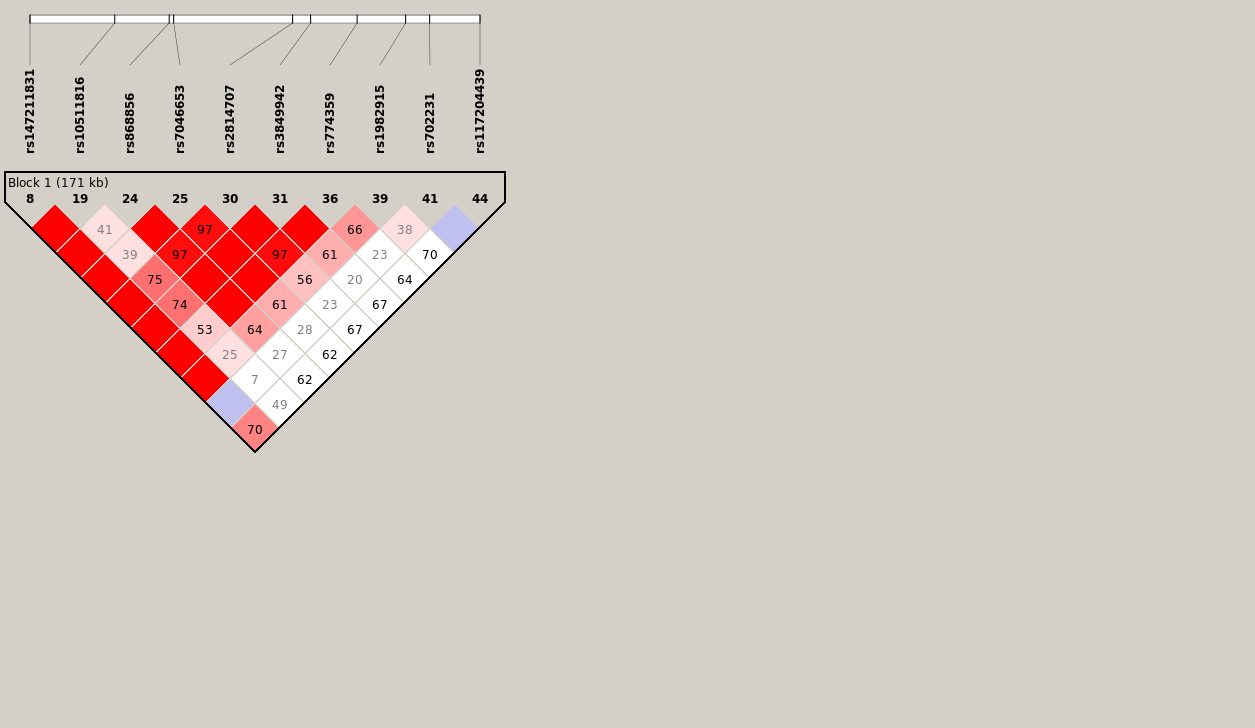

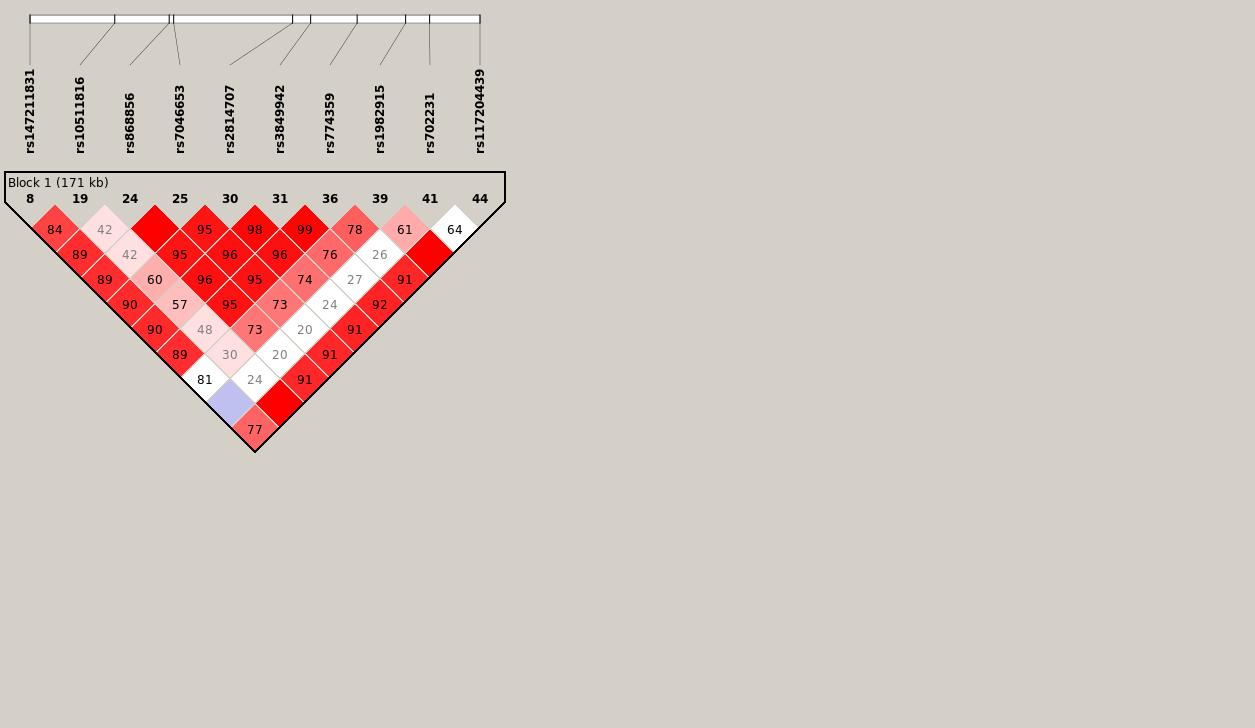


**Figure S1**. Phased SNPs entered into Haploview show that SNPs surrounding the *C9ORF72* gene form ‘one core haplotype’ block in patients with frontotemporal dementia (left) and control subjects (right).

As such, rs3849942 was used as proxy for the founder haplotype of the pathological *C9ORF72* repeat lengths. This is in accordance with previous studies and due to the tight linkage within the haplotype (Mok et al., 2012; Smith et al., 2013).

Abbreviation(s) SNPs: single nucleotide polymorphisms, *C9ORF72*: chromosome 9 open reading frame 72.


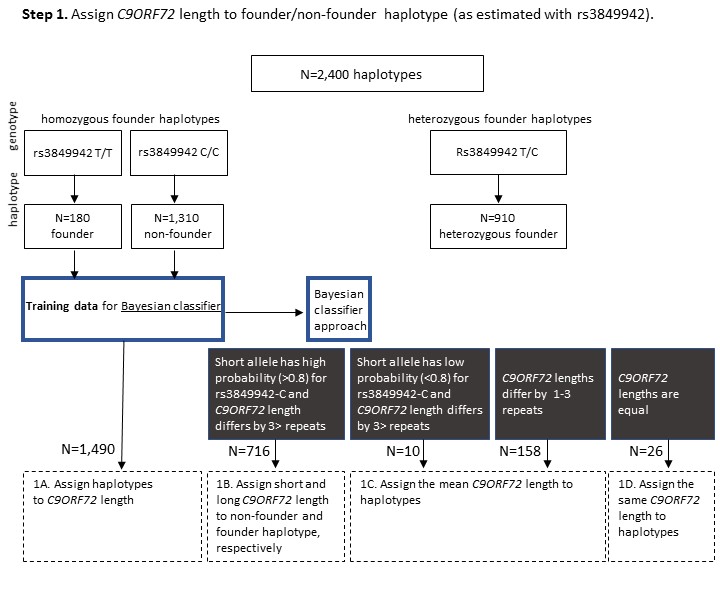


**Figure S2.** Overview of assignment of phased haplotypes to *C9ORF72* repeat length.

* Haplotypes with unsure risk haplotypes (N=66) are described in the supplementary materials.

Abbreviation(s) *C9ORF72*: chromosome 9 open reading frame 72.


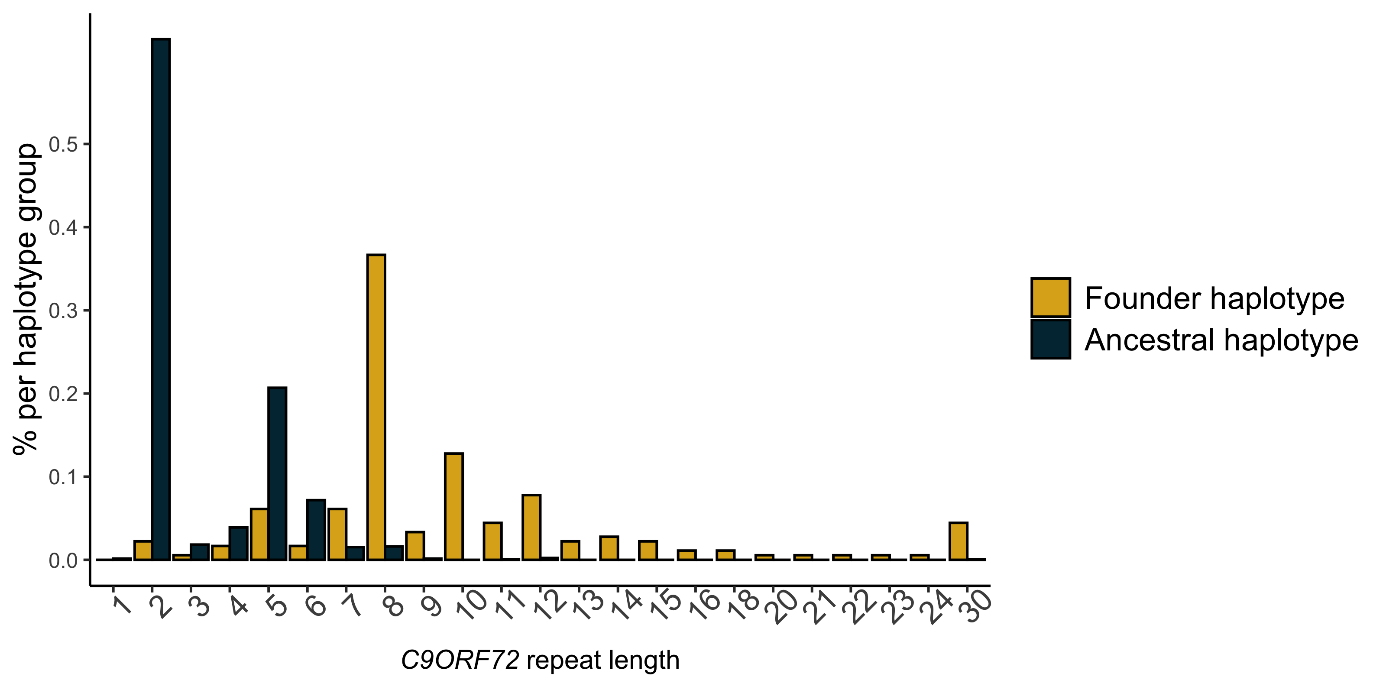


**Figure S3.** Frequency distribution of *C9ORF72* repeat lengths of N=1,310 ancestral (non-founder) and N=180 founder haplotypes.

The founder haplotype was estimated using rs3849942 (T-allele tags the founder haplotype). For this plot we only included haplotypes of individuals carrying two copies of the ancestral haplotype (rs3849942 C/C) or the founder haplotype (rs3849942 T/T).

Abbreviation(s) *C9ORF72*: chromosome 9 open reading frame 72.


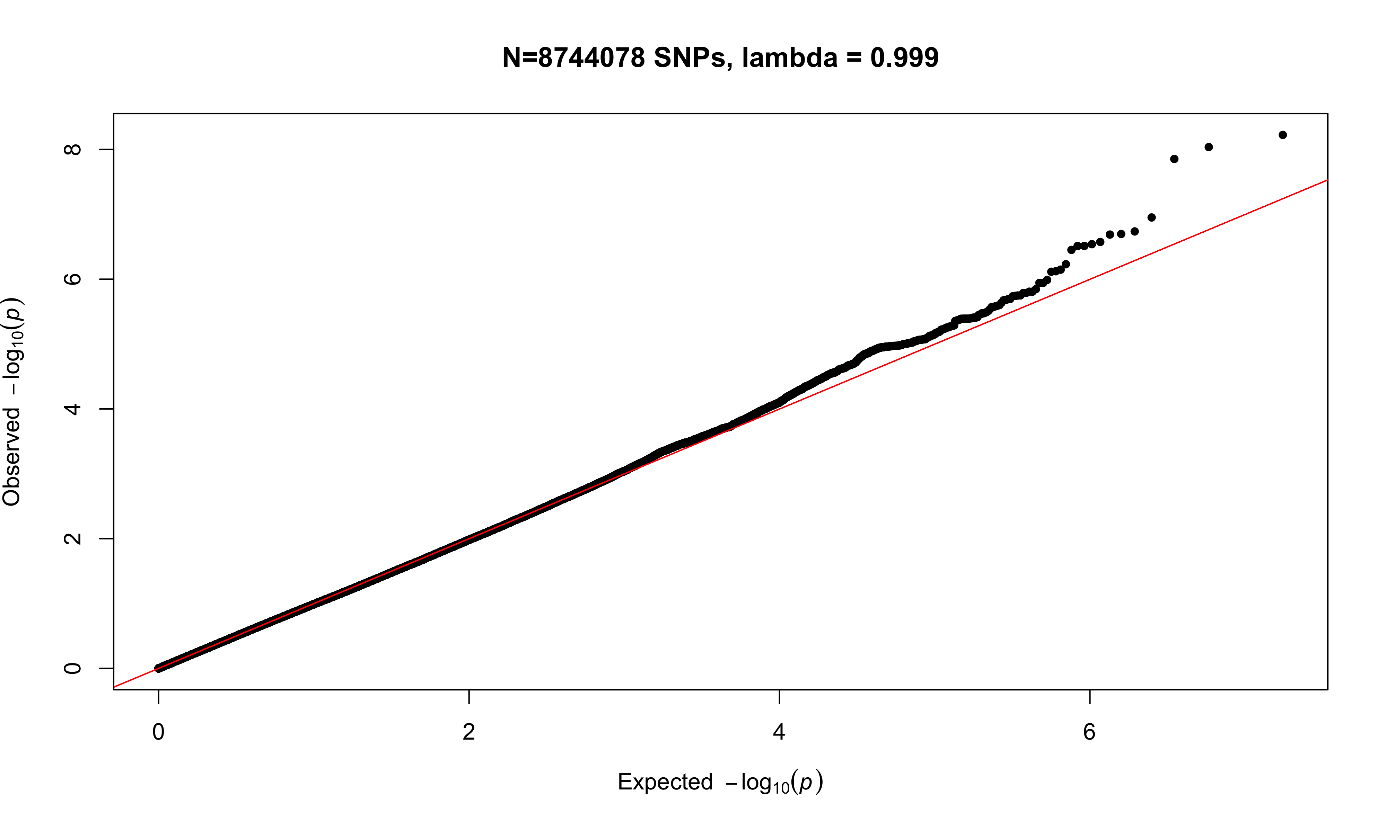


**Figure S4**. QQ-plot for the discovery analysis on the genome-wide association with frontotemporal dementia. No genomic inflation (λ=0.999) was observed.

**
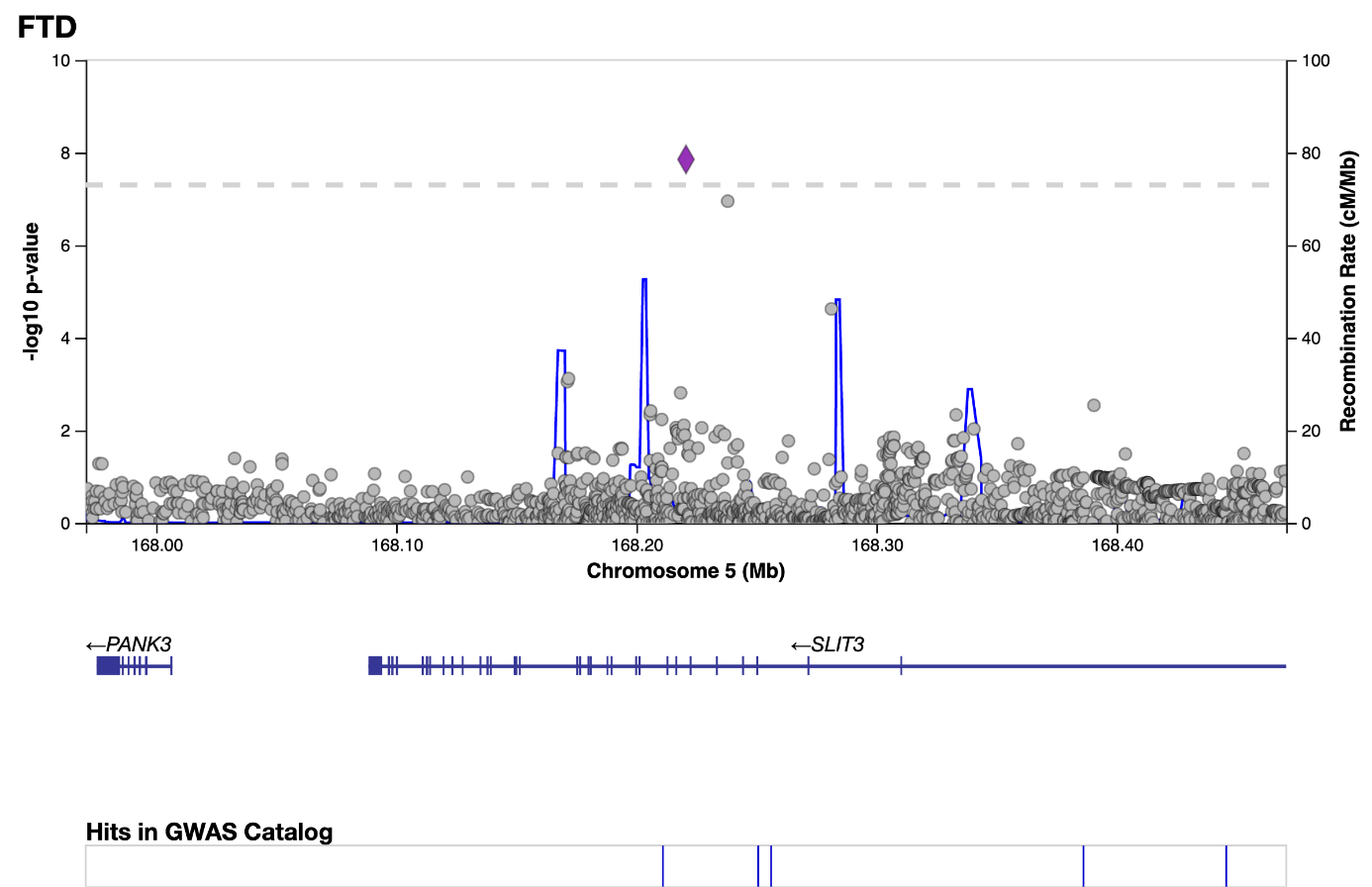
**

**Figure S5**. Regional plot for the *SLIT3* locus on chromosome 5.

The genetic variant depicted in purple represents the strongest associated variant.

Abbreviation(s) *SLIT3*: Slit Guidance Ligand 3.


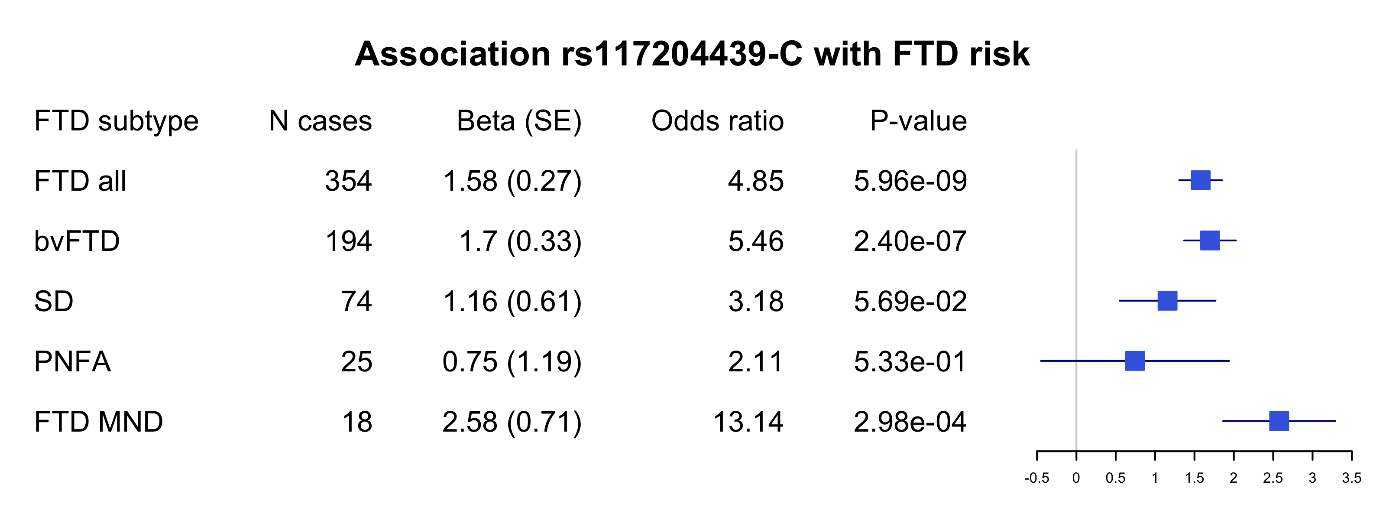


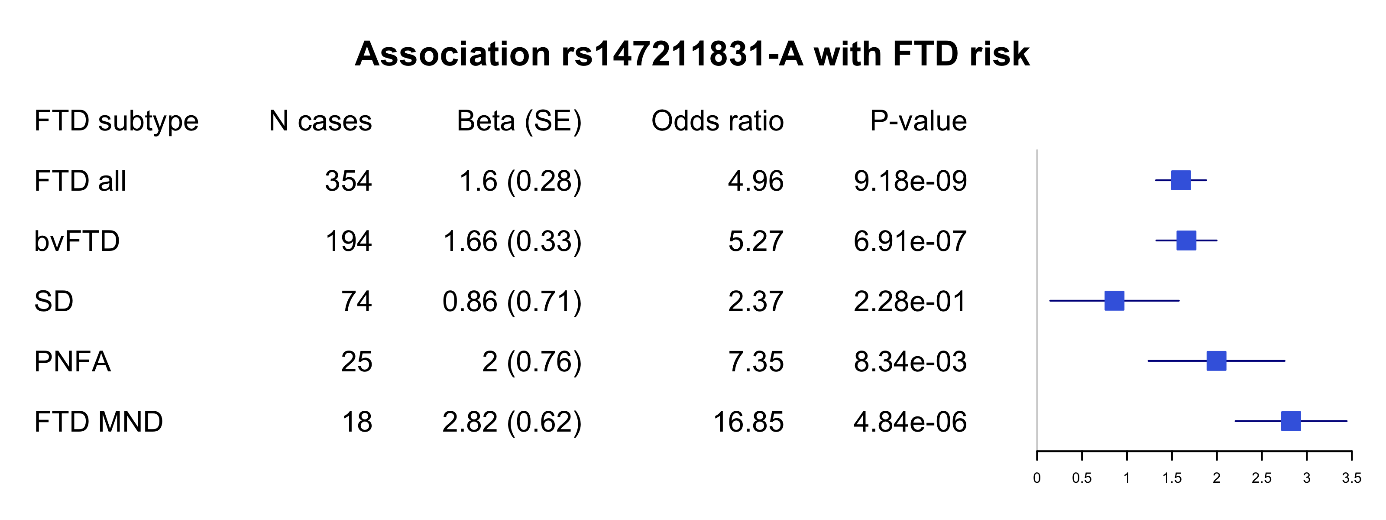


**Figure S6.** Forest plot of rs117204439-C (top) and rs147211831-A (bottom) on its genome-wide association with frontotemporal dementia and clinical frontotemporal dementia subtypes in the discovery analysis.

Abbreviation(s) FTD: frontotemporal dementia, bvFTD: behavioral variant of FTD, SD: semantic dementia, PNFA: progressive non-fluent aphasia, FTD-MND: FTD with motor neuron diseases, SE: standard error.

**Supplementary tables**

**Table S1.** Overview of studies included in the discovery or replication GWAS analysis on FTD.

Abbreviation(s) FTD: frontotemporal dementia, GWAS: genome-wide association study

**Table S2.** Demographic characteristics of FTD patients (N=327) and control subjects (N=4,209) included in the discovery analysis.

Abbreviation(s) FTD: frontotemporal dementia, bvFTD: behavioral variant frontotemporal dementia, SD: semantic dementia, PNFA: progressive non-fluent aphasia, FTD-MND: frontotemporal dementia with motor neuron disease, SD: standard deviation.

**Table S3.** FTD summary statistics discovery analysis adjusted for age and sex, and stratified by FTD clinical subgroups for SNPs rs147211831 and rs117204439.

Abbreviation(s) FTD: frontotemporal dementia, SNPs: single nucleotide polymorphisms, bvFTD: behavioral variant of FTD, SD: semantic dementia, PNFA: progressive non-fluent aphasia, FTD-MND: FTD with motor neuron diseases, ref: reference allele, alt: alternative allele, OR: odds ratio, Se: standard error.

**Table S4.** FTD summary statistics replication analysis stratified by FTD clinical subgroups for replicated SNPs rs147211831 and rs117204439.

Abbreviation(s) FTD: frontotemporal dementia, SNPs: single nucleotide polymorphisms, bvFTD: behavioral variant of FTD, SD: semantic dementia, PNFA: progressive non-fluent aphasia, FTD-MND: FTD with motor neuron diseases, ref: reference allele, alt: alternative allele, OR: odds ratio, Se: standard error.

**Table S5.** FTD summary statistics meta-analysis for replicated SNPs rs147211831 and rs117204439.

Abbreviation(s) FTD: frontotemporal dementia, SNPs: single nucleotide polymorphisms, ref: reference allele, alt: alternative allele, OR: odds ratio, Se: standard error.

**Table S6.** Identified risk variants for FTD show a significant association with ALS.

Abbreviation(s) FTD: frontotemporal dementia, ALS: amyotrophic lateral sclerosis, SNPs: single nucleotide polymorphism, Chr: chromosome, Bp: base pair location, ref: reference allele, alt: alternative allele, Se: standard error, *C9ORF72*: chromosome 9 open reading frame 72

**Table S7.** Demographics subjects included in the *C9ORF72* haplotype analysis.

N=4 missing values for sex status, N=10 missing values for age.

Abbreviation(s) *C9ORF72:* chromosome 9 open reading frame 72, DLB: dementia with Lewy bodies, FTD: frontotemporal dementia, MCI: mild cognitive impairment, AD: Alzheimer’s disease, PPA: primary progressive aphasia, SCD: subjective cognitive decline, SD: standard deviation.

**Table S8.** Group comparisons in *C9ORF72* repeat length between haplotypes.

Abbreviation(s) *C9ORF72*: chromosome 9 open reading frame 72, IQR: interquartile range.

**Table S9.** Group comparisons in *C9ORF72* length between founder and non-founder haplotypes for which three-SNP haplotypes could not be assigned to *C9ORF72* repeat lengths.

Abbreviation(s) *C9ORF72*: chromosome 9 open reading frame 72, IQR: interquartile range.
